# Bronchoscopic treatment of multiple bronchial myelolipomas: a case report and literature review

**DOI:** 10.1186/s12890-023-02608-z

**Published:** 2023-08-31

**Authors:** Jiali Ji, Hongqin Zhong, Xian Ren, Ting He, Guijuan Xie, Xun Wang

**Affiliations:** Department of Respiratory and Critical Care Medicine, Wuxi Second People’s Hospital, Jiangsu, China

**Keywords:** Myelolipoma, Bronchoscopy, Pulmonary atelectasis, Pneumonia

## Abstract

**Background:**

Extra-adrenal myelolipoma is an unusual entity, and endobronchial myelolipoma is rarer, which is often ignored by clinicians, delaying the disease and affecting the prognosis.

**Case presentation:**

A 71-year-old man with a history of chronic obstructive pulmonary disease (COPD) and type 2 diabetes mellitus, with recurrent fever, cough, and expectoration for more than 2 weeks experienced relief in cough, phlegm reduction, and glycemic control with anti-inflammatory treatment. Further examination revealed that new growths obstructing all lobar bronchi impaired flexible bronchoscope entry. In order to relieve the patient’s symptoms, under general anesthesia, we performed liquid nitrogen cryobiopsy at multiple bronchial openings, and then used argon plasma coagulation (APC) to achieve hemostasis. The pathological diagnosis was bronchial myelolipoma. The largest volume of the resected tissue was a mass measuring 0.6 cm × 0.4 cm × 0.3 cm at the bronchial opening of the upper lobe of the left lung. The patient’s condition was stable and the symptoms were partially relieved after surgery. No recurrence was observed during the 12-month follow-up, although the long-term treatment efficacy is unknown.

**Conclusion:**

Pathological biopsy is key to the diagnosis of endobronchial myelolipoma, and the development of the endobronchial myelolipomas may have been associated with long-term poor control of steroid levels in this patient.

## Background

Myelolipoma is a rare benign tumor, composed of mature adipose tissue and bone marrow hematopoietic tissue, and is commonly found in the adrenal glands [1]. The majority of adrenal myelolipomas are detected inadvertently on autopsy or surgically removed specimens [2]. Although the exact pathogenesis of adrenal myelolipoma is unknown, elevated adrenocorticotropic hormone (ACTH) levels and sustained ACTH stimulation (e.g., Congenital Adrenal Hyperplasia and Cushing’s disease) likely play an important role [3–5].

Extra-adrenal myelolipomas are rarer, and account for only 15% of myelolipomas, of which 3% occur in the chest, mostly in the posterior mediastinum [6]. Myelolipomas can occur in the liver, spleen, lung, and other extra-adrenal sites, as reported recently [7, 8]. However, only 18 cases of pulmonary myelolipomas have been reported, both at home and abroad [9–15]; among these, two cases involved only the bronchus and not lung parenchyma [12].

We hereby present the case of treatment of multiple bronchial myelolipomas, along with a literature review of similar cases.

## Case presentation

A 71-year-old Chinese man presented with recurrent fever and persistent cough, sputum, and asthma 2 weeks. Antibiotic treatment in the community hospital initially led to symptom alleviation, but was followed by a relapse, and the patient came to our hospital for treatment. The patient had type 2 diabetes and chronic cholecystitis with gallstones and was receiving subcutaneously injected recombinant insulin glargine (7 units QN) and insulin aspart (7 units TID), and oral acarbose and sitagliptin, but without adequate glycemic control. In the past 20 years, the patients had experienced recurrent cough, expectoration, and asthma, usually in autumn and winter. In the past 3 years, the patient was diagnosed with exertional dyspnea, and the symptoms improved after each antibiotic treatment. The patient has a long history of smoking for nearly 40 years.

The patient had a barrel chest and slight dyspnea. Percussion of both lung fields elicited hyperresonance. Auscultatory breath sounds were muffled, and widespread wheezing and rales were heard without pleural rubbings.

**Fig. 1:**
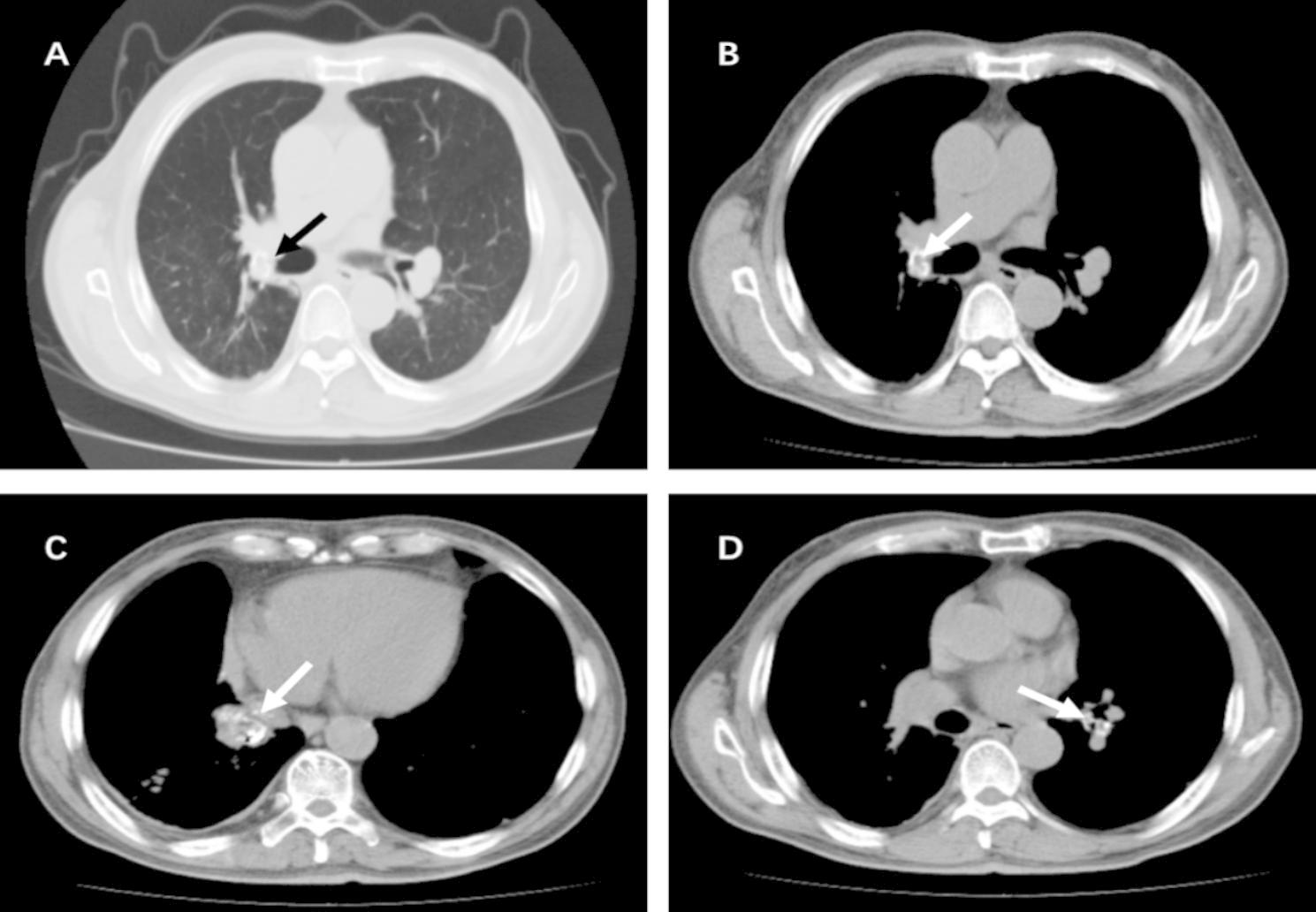
CT findings of multiple endobronchial masses. **(A and B)** Lung window and mediastinal chest CT scanning images showed an endobronchial mass (arrow) that obstructed the bronchi in the right upper lobe of the lung. **(C)** Mediastinal window showed a mass (arrow) that blocked the bronchi in the lower lobe of the right lung. **(D)** Mediastinal window showed obstruction (arrow) in the lower lobe of the left lung

Laboratory examinations that were conducted at admission included routine blood investigation that revealed WBC 14.7 × 10^9^/L (neutrophile granulocyte: 85.8%). Sputum smear examination did not reveal fungal, bacterial, mycobacterial, or other infection. The T-spoT test showed a negative result. Despite a normal glycated hemoglobin level, the fasting plasma glucose remained approximately 234 mg/dL. CT of the chest revealed multiple high-density pulmonary masses bilaterally in the bronchial openings of the upper, middle and lower lobes, accompanied by bronchiectasis (Fig. 1). Access for fiberoptic bronchoscopy was limited as new growth obstructed the bronchoscope lumen at all lobar bronchi. We performed forceps biopsy, brushing, and alveolar lavage at multiple bronchial openings in batches. Examination of the bronchoscopic brushing and lavage fluid revealed ciliated columnar and bronchial mucosal epithelium, respectively, but no signs of carcinoma. Pathological examination of the specimen revealed chronic inflammation of the mucosa, trabecular bone structure with calcification, fat, and neovascularization. Due to the limited amount of tissue obtained by forceps biopsy, the pathologic diagnosis only demonstrated a non-specific airway tumor. The specific diagnosis required further bronchoscopic pathological biopsy.

**Fig. 2:**
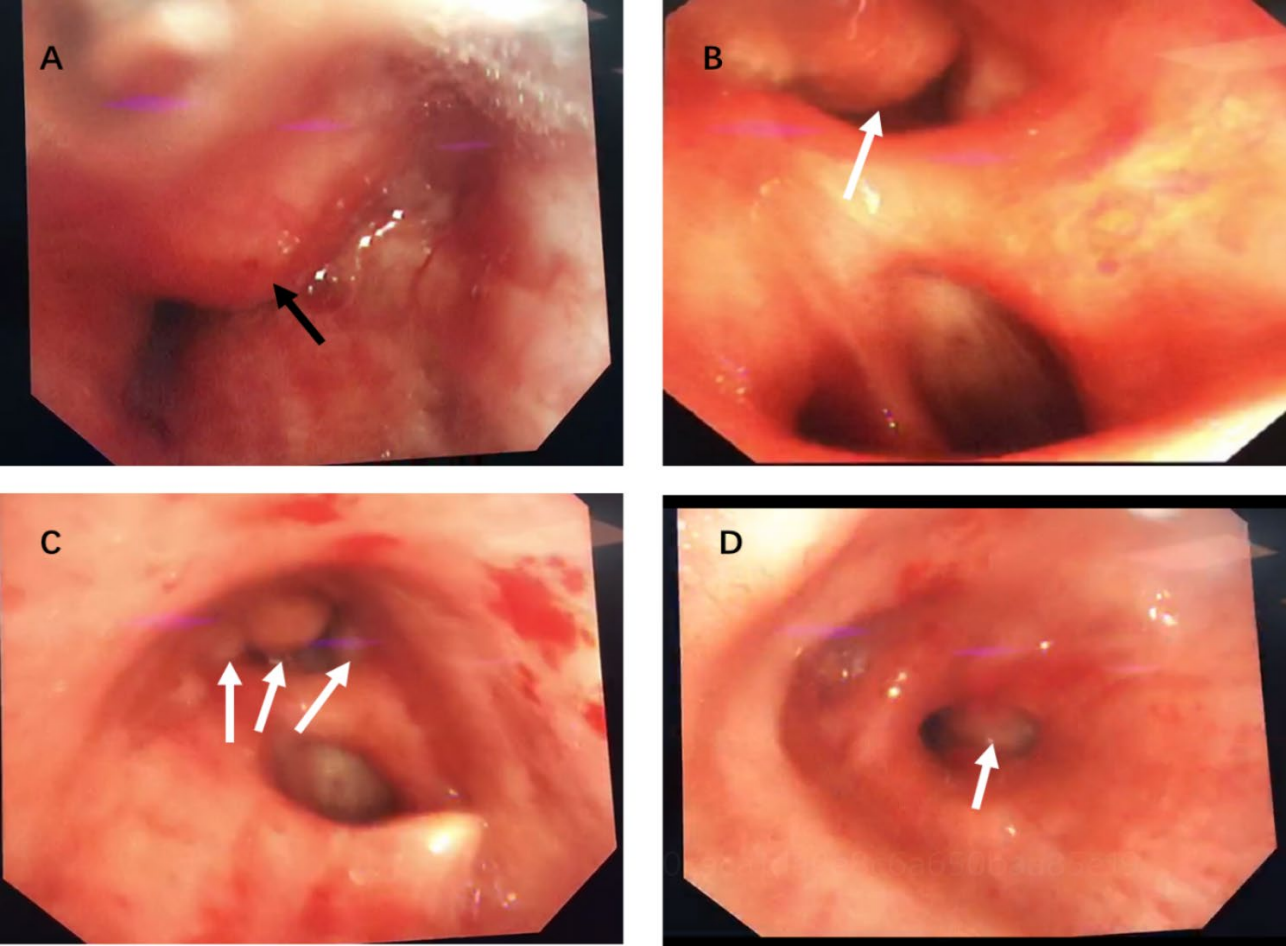
Bronchoscopic images of some lesions. **(A and B)** Neoplasms in the bronchi of the left upper and lower lobes, with smooth surface and occluded lumen that obstructed the bronchoscope entry (black arrow indicates the site of the operation). **(C and D)** New growths in the openings of the right upper, middle, and lower lobes, that had a smooth surface and partially blocked the lumen

To obtain definitive diagnosis and effective treatment, we scheduled bronchoscopic tumor resection and cryotherapy under general anesthesia again. The multiple bronchial openings were reluctantly selected for a liquid nitrogen cryobiopsy (Fig. 2). Then APC was used to ensure hemostasis.

**Fig. 3:**
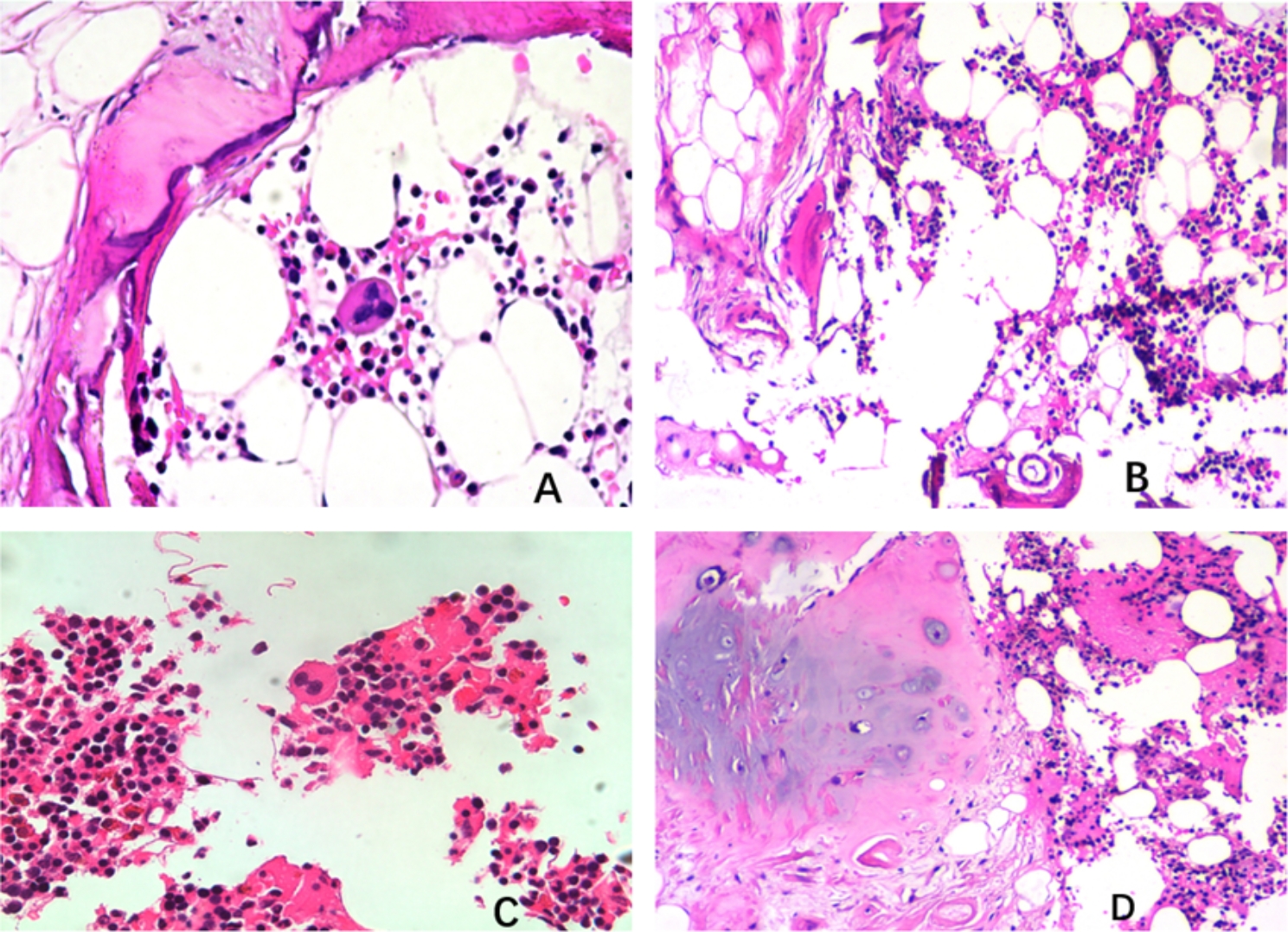
Hematoxylin-eosin staining (HE staining) results. **(A)** Trabecular bone, semicircular and c-type enclosing tumor cells; magnification ×400. **(B)** Hematopoietic cells were seen in mature adipose tissue; magnification ×200. **(C)** Hematopoietic cells of erythrocyte, granulocyte, and megakaryocyte lineage; magnification ×400. **(D)** Trabeculae are seen in some areas; magnification ×400

**Fig. 4:**
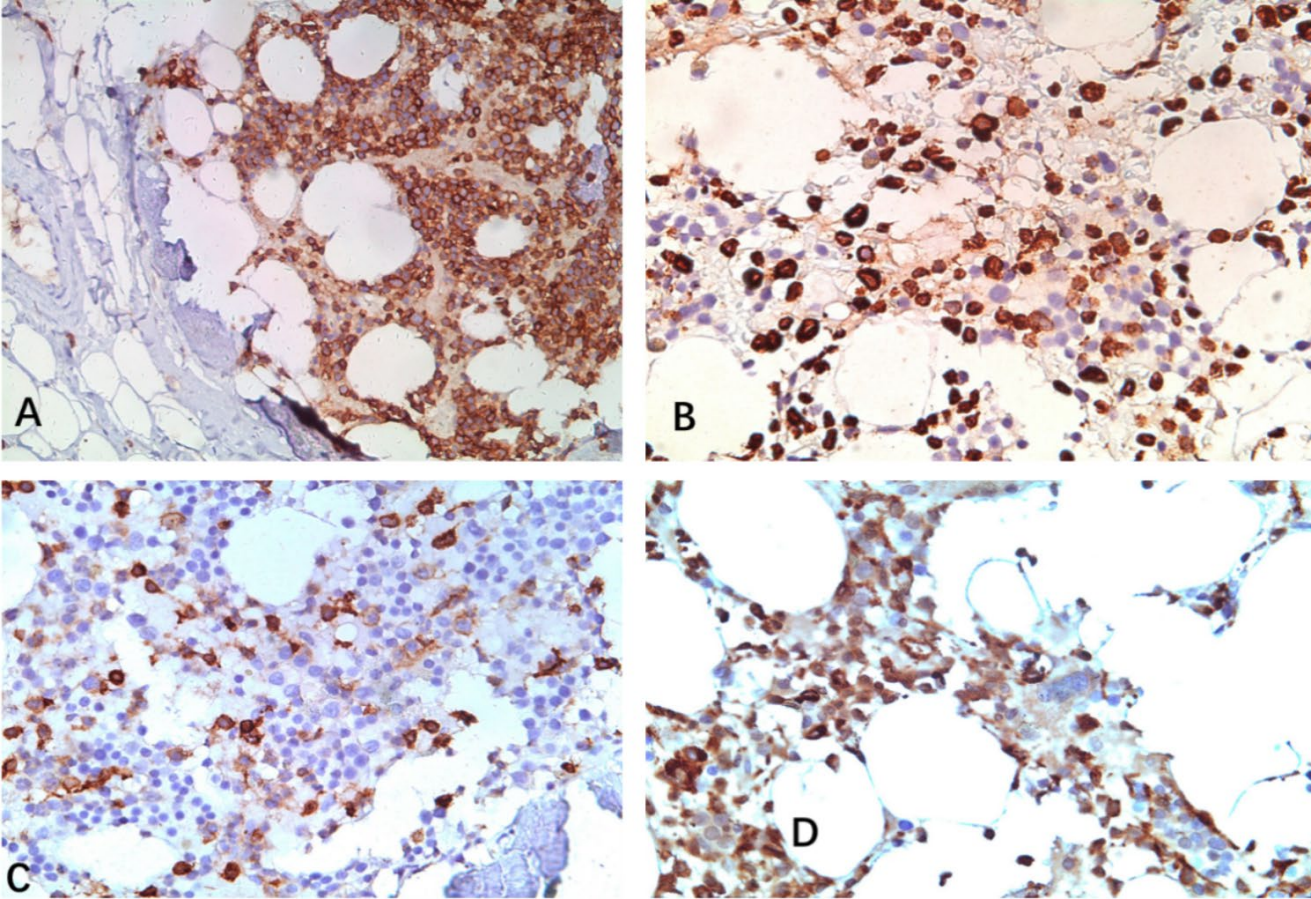
Immunohistochemical findings. **(A)** Diffuse positive expression of LCA in tumor cells. **(B)** Positive expression of CD3 on lymphocytes. **(C)** Positive expression of FVIII in the megakaryocyte cell lineage. **(D)** Positive MPO expression in granulocytes; magnification ×200

A relatively large piece of tissue taken from the bronchial opening of the upper lobe of the left lung can be seen roughly 0.6 cm × 0.4 cm × 0.3 cm grayish red with slightly hard cut texture. Microscopically, ciliated columnar epithelium covered the surface, and multifocal nodular tumor areas were seen in the fibrous tissue. The nodules were surrounded by semicircular and c-shaped trabecular bone. The tumor cells were mainly aggregated granulocytes, erythrocytes, and scattered multinucleated giant cells that were distributed among mature adipocytes (Fig. 3). Immunohistochemistry revealed a mix of megakaryocytic (CD61, FVIII, CD68, LCA, CD3, CD20, CD43) and granulocytic (MPO) origin. (Fig. 4). These results support the definite diagnosis of myelolipoma. Postoperatively, the patients experienced slight alleviation of the airway stenosis and the symptoms of infection were effectively controlled. Postoperative contrast-enhanced CT showed that the state of inflammation and atelectasis had subsided although they had not completely disappeared. Subsequently, the patient actively fought infection and controlled blood glucose, and the symptoms improved further. Although the patient’s condition was slightly reduced in terms of symptoms and examination results, multiple bronchial masses still existed, so the prognosis of the patient remains guarded.

## Discussion

Myelolipomas were first reported in the early 20th century and were mainly related to the adrenal gland. Extra-adrenal myelolipomas are rare, and pulmonary myelolipomas are rarer. Thus far, only 18 cases of pulmonary myelolipoma have been reported at home and abroad, and we report the 19th case. Table 1 summarizes the clinicopathological features of all 19 cases: 14 (73.7%) patients were male and only 5 (26.3%) were female. The vast majority of patients were middle-aged or older adults, and the youngest patient was 29 years old. Most of the patients presented with pneumonia or other systemic diseases, and only one patient had a history of lung cancer. Pulmonary myelolipoma was often misdiagnosed because it can only be detected by surgical excision, pathological examination, and autopsy. Only two cases involved solely the bronchus, without invading the lung parenchyma, but unlike in our patient, both involved a single mass.

**Table 1:** Patient and tumor characteristics of reported intrapulmonary myelolipomas

Case	First author/s	Age/sex	Patient history	Tumor location	Number of sizes	Size, cm	Diagnostic method
1	Saleeby	81/F	Pneumonia	Peripherally	Single	Φ2.5	Autopsy
2	Hunter et al.	70/F	RA, steroid	Peripherally	Multiple	No measured	Biopsy
3	Ziókowski et al.	49/M	Pneumonia	LLL	Multiple	7 × 5 × 5	Autopsy
4	Ziókowski et al.	59/M	/	RLL	Single	Φ2.0	Resection
5	Zunarelli et al.	52/M	BC	RLL	Single	No measured	Lobectomy
6	Sabate and Shahian	54/M	HC	LUL	Single	Φ2.5	Enucleation
7	Lu et al.	45/M	Pneumonia	LUL	Single	Φ1.5	Lobectomy
8	Sato et al.	71/M	Lung cancer	LUL	Single	Φ2.0	Autopsy
9	Lin et al.	45/M	/	LUL	Single	4.5 × 3.5 × 2.3	/
10	Liu et al.	63/M	/	LMB	Single	2 × 1 × 0.5	/
11	Xing et al.	61/M	Asymptomatic	RML	Single	Φ1.5	/
12	Huang et al.	53/M	Pneumonia, Atelectasis	LLL	Single	2.3 × 1.2 × 1.0	Lobectomy
13	Huang et al.	57/F	Bronchiectasis	RLL	Single	Φ1.6	Lobectomy
14	Silvija et al.	83/F	/	RLL	Single	Φ3.0	Autopsy
15	Chung et al.	38/M	Pneumonia	RB	Single	1.3 × 2.0	Bronchoscopy
16	Wang et al.	49/F	Asymptomatic	RLL	Single	2.5 × 2.0 × 2.0	/
17	Zhan et al.	61/M	Asymptomatic	LLL	Single	1.4 × 1.3	Resection
18	Zhang et al.	29/M	Cervicodynia	RLL	Single	7 × 4 × 3	Resection
19	Present case	71/M	Pneumonia	Lobar bronchi	Multiple	0.6 × 0.4 × 0.3	Bronchoscopy

*F, female; M, male; RA, rheumatoid arthritis; BC, bronchial carcinoid tumor; HC, hypercholesterolemia; LLL, left lower lobe; LUL, left upper lobe; RLL, right lower lobe; RUL, right upper lobe; LMB, left main bronchus; RML, right main lobe; RB, right bronchus.

The etiology of myelolipoma is not fully understood, although several theories have been propounded, of which the most widely accepted one is that the myelolipoma may originate in embryonal mesenchymal cells of the adrenal gland. Under certain conditions (e.g., tissue injury, chronic infection, abnormal endocrine hormone levels, etc.), these interstitial cells can not only differentiate into bone marrow hematopoietic tissue but also undergo metaplasia into adipose tissue [16]. Patients with adrenal myelolipoma often have abnormal corticosteroid levels (with attendant obesity, hypertension, diabetes, etc.) or some syndromes that result in endocrine abnormalities (e.g., Conn syndrome, Cushing syndrome, etc.) [3–5], [17, 18]. Our patient had a history of type 2 diabetes with chronically poor glycemic control, which is aligned with the theory of hormonal disorders. However, the relationship between pulmonary myelolipoma and neuroendocrine dysregulation has not been reported previously. Therefore, we hypothesize that, similar to adrenal myelolipoma, endobronchial myelolipoma production may be influenced by steroids.

Histologically, myelolipoma consists of mature adipose tissue and hematopoietic tissues, including myeloid cells, erythrocytes, megakaryocytes, and, occasionally, lymphocytes. The presence of bone tissue in myelolipoma remains controversial. Our literature review revealed that trabecular or segmental bone was found in 8 of the 19 cases [10, 11, 13, 14]. A study found that a bony component of pulmonary myeloma may be related to the site of tumor occurrence, especially near the bronchial cartilage, as in our case [6].

Pathological diagnosis is the gold standard for the diagnosis of endobronchial myelolipoma, which is often similar to the pathological features of the following diseases and needs to be differentiated. (1) Extramedullary hematopoiesis: extramedullary hematopoiesis often occurs in the spleen, rarely in the lung, and often accompanied by hematological diseases [19, 20]. It is usually a diffuse and scattered small focus of bone marrow hematopoietic tissue with unclear boundary. Under the microscope, it is usually dominated by red blood cells without adipose tissue. (2) Lipoma: endobronchial lipoma is a rare benign lung tumor that can cause bronchial obstruction and parenchymal injury. A large amount of adipose tissue without hematopoietic tissue can be seen under the microscope. (3) Hamartoma: mainly occurs in the peripheral lung, < 10% occurs in the bronchus. Unlike myelolipoma, it is composed of a mixture of cartilage, fiber, fat, smooth muscle, and blood vessels without hematopoietic components [21]. (4) Ectopic bone marrow: the tissue often contains calcification and more medullary trabeculae, which is helpful to distinguish it from myelolipoma.

Benign endobronchial neoplasms are particularly rare clinical entities. Bronchoscopy is often used for palliative treatment of intrabronchial malignancies [22–24]. However, for several years, the use of bronchoscopy to remove benign lesions has been reported [25–27]. Cryotherapy or thermal ablation in the airway to treat airway diseases, including benign or malignant central airway obstruction (CAO), has become increasingly popular and is replacing traditional surgery. Common techniques include electrocautery, balloon expansion (BD), neodymium doped: yttrium aluminum garnet (Nd: YAG) laser, APC, and cryotherapy, which may usually be used in combination to confer greater effect [28].

We chose APC in combination with cryotherapy based on the location of the patient’s mass. APC is a non-contact technology that uses ionized argon (plasma) to apply a monopole current to the nearest tissue with a penetration depth of less than 3 mm. APC is very safe and is mainly used to treat superficial flat lesions [29, 30]. As argon can flow over angles and into corners, APC is suitable for treating acute-angled bronchial segments (e.g., the apical and posterior segments of the upper lobe). However, the disadvantages of APC are obvious: eschar forms easily and needs to be removed with biopsy forceps. APC, in combination with cryotherapy, enables rapid removal of intrabronchial masses, shortens the operative time, and decreases the risk of major bleeding [31].

## Conclusion

We diagnosed and treated a patient multiple bronchial myelolipoma that did not involve the lung parenchyma. Tissue sections showed bony tissue, bone trabeculae, and ossification. The patient had slight symptom improvement with bronchial interventional therapy, APC, and cryotherapy. However, the relationship between pulmonary myelolipoma and neuroendocrinology has not been clearly explained, and deserves further study.

## Data Availability

All data and materials are provided in the manuscript.

## Abbreviations

QN: Once a night
TID: three times a day
WBC: White blood cell
T-spoT test: T cell spot test of tuberculosis infection
CT: Computerized tomography
LCA: leukocyte common antigen
MPO: myeloperoxidase
HE staining: hematoxylin-eosin staining
FVIII: Factor VIII-related antigen
